# Idiopathic Peripheral Retinal Telangiectasia in Adults: A Case Series and Literature Review

**DOI:** 10.3390/jcm10081767

**Published:** 2021-04-19

**Authors:** Maciej Gawęcki

**Affiliations:** Dobry Wzrok Ophthalmological Clinic, 80-822 Gdansk, Poland; maciej@gawecki.com; Tel.: +48-501-788-654

**Keywords:** Coats disease, peripheral retinal telangiectasia, laser photocoagulation, anti-VEGF treatment

## Abstract

Idiopathic peripheral retinal telangiectasia (IPT), often termed as Coats disease, can present in a milder form with the onset in adulthood. The goal of this case series study and literature review was to describe and classify different presenting forms and treatment of this entity and to review contemporary methods of its management. Six cases of adult onset IPT were described with the following phenotypes based on fundus ophthalmoscopy, fluorescein angiography, and optical coherence tomography findings: IPT without exudates or foveal involvement, IPT with peripheral exudates without foveal involvement, IPT with peripheral exudates and cystoid macular edema, and IPT with peripheral and macular hard exudates. Treatments applied in this series included observation, laser photocoagulation, and anti-vascular endothelial growth factor (VEGF) treatment with variable outcomes depending upon the extent of IPT, the aggressiveness of laser treatment, and the stringency of follow-up. The accompanying literature review suggests that ablative therapies, especially laser photocoagulation, remain the most effective treatment option in adult-onset IPT, with anti-VEGF therapy serving as an adjuvant procedure. Close follow-up is necessary to achieve and maintain reasonable good visual and morphological results.

## 1. Introduction

Idiopathic peripheral retinal telangiectasia (IPT), usually referred to as Coats disease, has been well-described in the medical literature since its discovery in 1908 [1]. Coats disease is an idiopathic condition characterized by telangiectatic and aneurysmal retinal vessels with intraretinal and subretinal exudation and fluid without appreciable retinal or vitreal traction, frequently associated with retinal detachment [2]. The pathogenesis and progression of that disease depends on the impairment of retinal vasculature. Alterations of endothelium of retinal vessels lead to breaking of blood-retinal barrier and presence of abnormal pericytes, causing formation of telangiectasias and closure of some vessels [3,4]. Exudation of lipids from the damaged vessels follows and is responsible for retinal detachment and cyst formation as well as retinal ischemia due to vessel closure [5]. The process is described as similar to pathogenesis of diabetic retinopathy, but definitely more severe and rapid [6].

Coats disease commonly presents unilaterally with strong male predominance. It has reportedly occurred in patients ranging from 4 months to 70 years of age, with the peak incidence in the first decade of life [5]. Its severe form (referred to as Coats disease) and milder form (sometimes termed Leber miliary aneurysms) that commonly present in adults, were initially described as separate entities [7]. Now, they are considered to be variable expressions of the same disease This explains its adult onset, as milder forms often stay asymptomatic for a long period of time [8,9]. Less severe forms of Coats disease fall into category 1, 2A and 2 B of the Shields classification commonly used to describe the stages of this disease [10]. Nevertheless, the perception of many ophthalmologists is that Coats disease is attributed to young children, only, and limited peripheral forms of this clinical entity are still frequently labeled differently, which causes inconsistencies in classification of the disease. Moreover, different systems of classification of retinal telangiectasia sometimes mix peripheral and central variants of the entity [11,12]. Terminology used to describe retinal telangiectasias together with their characteristics is provided in Table 1.

The diagnosis of IPT also can be challenging. Peripheral retinal telangiectasias accompany some rare systemic conditions, so before diagnosing the idiopathic form, it is important to rule out the primary underlying condition. Moreover, IPT is often asymptomatic and, as such, frequently missed, thus obscuring the real prevalence of the disease. On the other hand, if IPT results in macular edema and the far periphery of the retina is not examined, the condition might not be recognized as being secondary to the lesion located outside the posterior pole, leading to an erroneous therapeutic approach and sometimes lack of medical improvement.

Finally, there are numerous approaches to treatment for the different phenotypes of IPT, which have evolved in concert with progress in ophthalmology and the advent of new therapeutic methods.

The goal of this case series report was to present the different forms of presentation of IPT seen in an adult patient population as well as the observed responses to variable treatment modalities. Various diagnostic and therapeutic scenarios experienced by the author are placed in the perspective of available research from other clinicians.

## 2. Materials and Methods

A retrospective search of Dobry Wzrok clinic medical records was conducted to identify adult patients with peripheral telangiectasia confirmed by the results of fluorescein angiography (FA). Cases of aneurysmal macular telangiectasia (MACTEL type 1) as described in the classification of MACTEL proposed by Yanuzzi in 2006 were excluded from this study because they involved a centralized lesion location [13]. Additionally, a literature search was performed in the PubMed database for the phrase “peripheral retinal telangiectasias” and the combination of words “Coats disease” and “adult onset.”

## 3. Results

The Dobry Wzrok clinic records contained six cases of idiopathic peripheral retinal telangiectasia in adults aged 21 to 60 years, including five men and one woman. In all cases, lesions were limited to one eye only and the other eye remained without compromise. Patient characteristics are presented in Table 2.

Study participants were divided into four categories depending on their presentation as follows:

- Category 1: IPT without peripheral exudates and without macular involvement (patient 1)
- Category 2: IPT with peripheral exudates and without macular involvement (patient 2)
- Category 3: IPT with peripheral exudates and cystoid macular edema without exudates (patients 3,4,5)
- Category 4: IPT with peripheral exudates and macular hard exudates and edema (patient 6)

### 3.1. Categories 1 and 2

**Figure 1:**
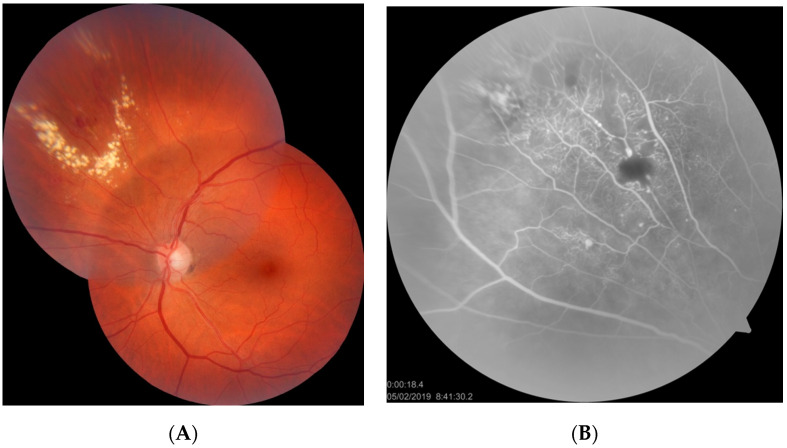
Fundus photograph (**A**) and FA image (**B**) of patient 2. A ring of hard exudates is visible at the periphery of the supranasal quadrant of the fundus. Fluorescein angiography revealed IPT located within the relatively small area of hard exudates. Macular edema is absent.

**Figure 2:**
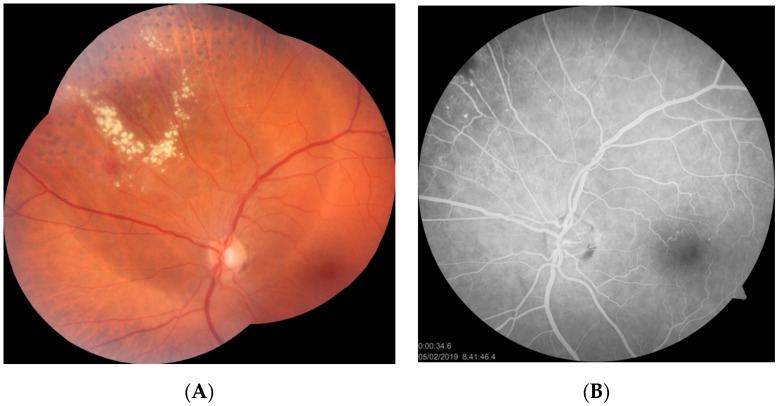
Fundus photograph (**A**) and FA image (**B**) of patient 2 after LPC of IPT. Partial resolution of hard exudates is visible.

Patients 1 and 2 were two cases of asymptomatic IPT found in our records search. In both cases, the length of follow-up was about 12 months. Both cases were asymptomatic—the lesions were found during routine ophthalmological examinations. Fundus examination and FA in both cases revealed IPT limited to a relatively small area of the peripheral retina. In one case, some amount of flat, hard exudates was observed at the border of the lesion, but macular edema was not present (patient 2) (Figure 1). In that case, laser photocoagulation (LPC) was applied to the IPT area (Figure 2). The other case was followed without any treatment; during the follow-up period, no telangiectasia progression was observed.

### 3.2. Category 3

**Figure 3:**
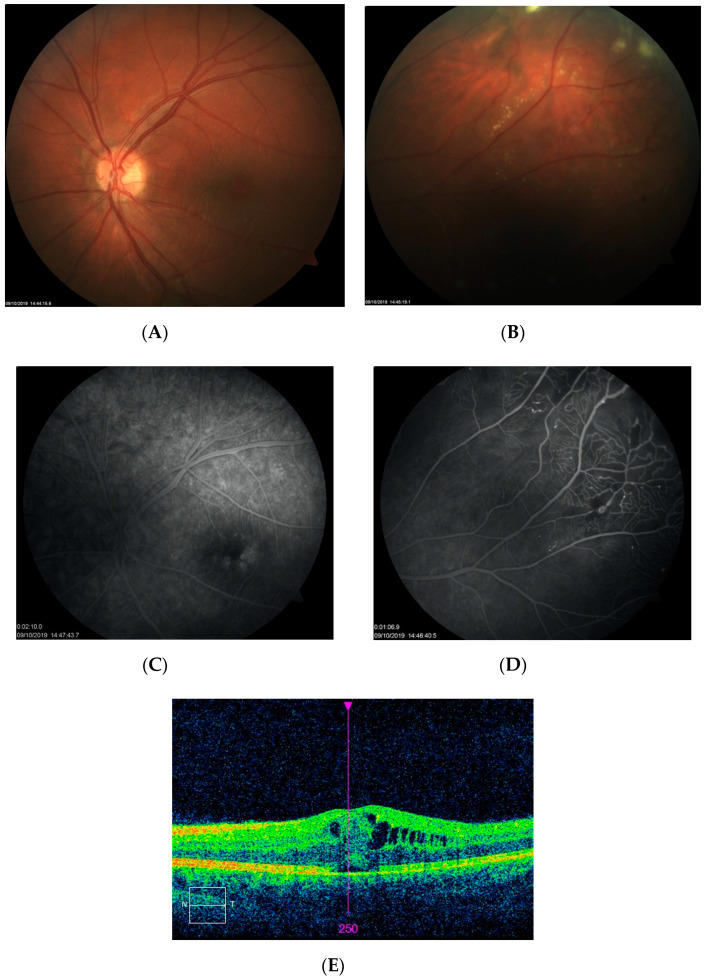
Pretreatment fundus photographs (**A**,**B**), FA images (**C**,**D**), and SD-OCT macular scan (**E**) of patient 3. Hard exudates are visible in supratemporal quadrant of the fundus of LE. FA reveals IPT and CME. CME is confirmed by SD-OCT scan.

**Figure 4:**
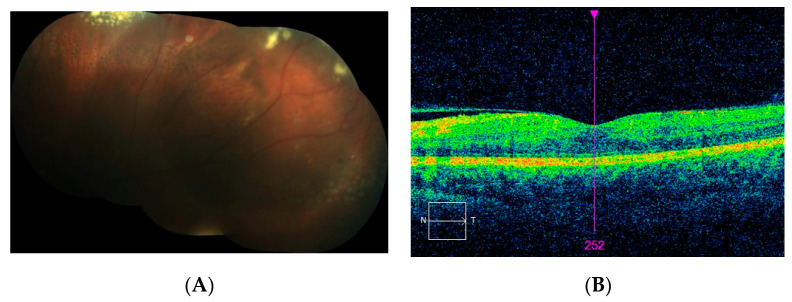
Fundus photograph (**A**) and SD-OCT macular scan (**B**) of patient 3 after LPC and intravitreal aflibercept therapy. Laser burns are visible on the color fundus photograph. Remission of CME is noted on the SD-OCT scans.

Category 3 consisted of three cases of IPT that presented with mild to moderate macular cystoid edema without hard exudates (patients 3–5). All of these patients were symptomatic and complained of vision impairment in one eye. In all cases, the duration of symptoms was relatively short, according to the patients, at between one and three months. The diagnosis of IPT was confirmed by FA and macular edema was evaluated and monitored with spectral-domain optical coherence tomography (SD-OCT). Two cases (patients 3, 4) were treated with LPC of peripheral lesions combined with intravitreal bevacizumab or aflibercept injections. One patient (patient 5) underwent LPC and was scheduled for anti-vascular endothelial growth factor (VEGF) treatment. In one case (patient 3), complete remission of CME was noted after 12 months of follow-up; his BCVA remained stable at the level of 20/30 and did not improve further. FA photographs and SD-OCT scans before and after treatment are presented in Figure 3 and Figure 4.

**Figure 5:**
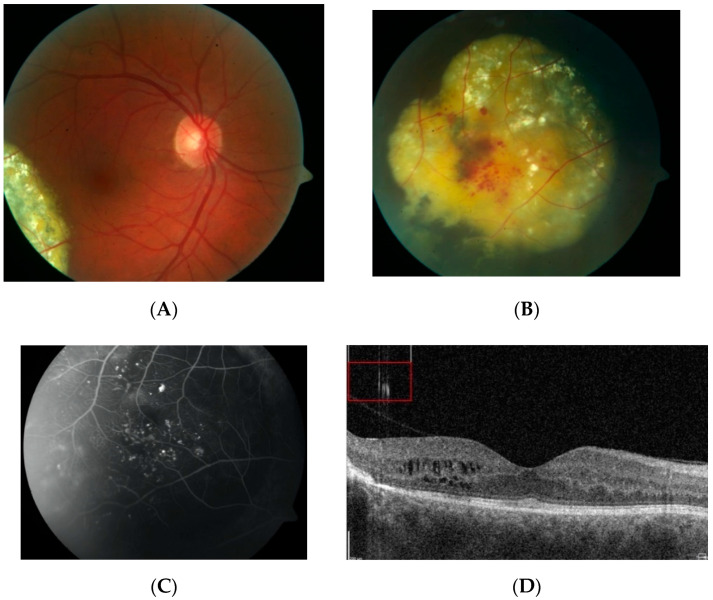
Fundus photographs (**A**,**B**), FA image (**C**), and SD-OCT scan (**D**) at presentation of patient 4. Color fundus photographs show a well-demarcated lesion located temporally of the macular area. FA reveals the presence of IPT within the area of the lesion. Only a mild CME is visible on the SD-OCT scan.

**Figure 6:**
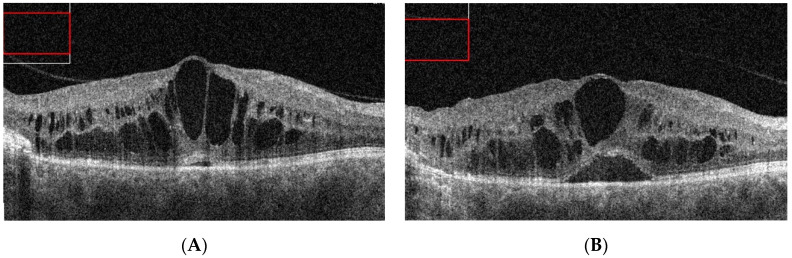
SD-OCT macular scans of patient 4 at 12 months after initial presentation (**A**) and after treatment with LPC and intravitreal anti-VEGF injections (**B**). Large CME with large pseudocysts is noted on the SD-OCT scans. Only a minor reduction of edema is observed after treatment.

Patient 4 from category 3 presented with IPT in the shape of a round, well-demarcated lesion bordering the temporal sector of the macular area and with the fovea appearing normal during the biomicroscopic examination. FA imaging revealed a typical IPT pattern within the lesion (Figure 5C). There was only a mild CME detected by SD-OCT, which was treated subsequently with subthreshold micropulse laser (Figure 5D). The patient did not show up for follow-up until his vision had significantly decreased to 20/100 at 12 months after his initial presentation. SD-OCT scan at that time revealed significant macular edema with defects at the ellipsoid zone (Figure 6A). LPC was used to create a line demarcating the lesion from the fovea in combination with intravitreal bevacizumab. Intravitreal injections were repeated several times over the following nine months, without significant morphological changes in the central retina or BCVA improvement (Figure 6B). At that point, other therapeutic options were considered, such as more intensive LPC or cryotherapy of the lesion.

**Figure 7:**
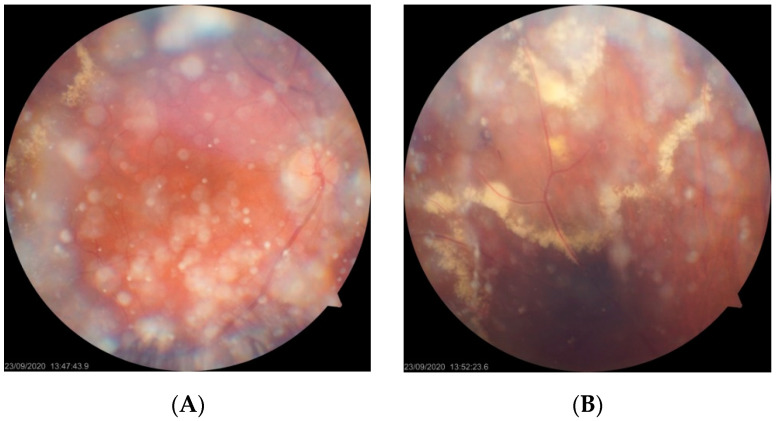

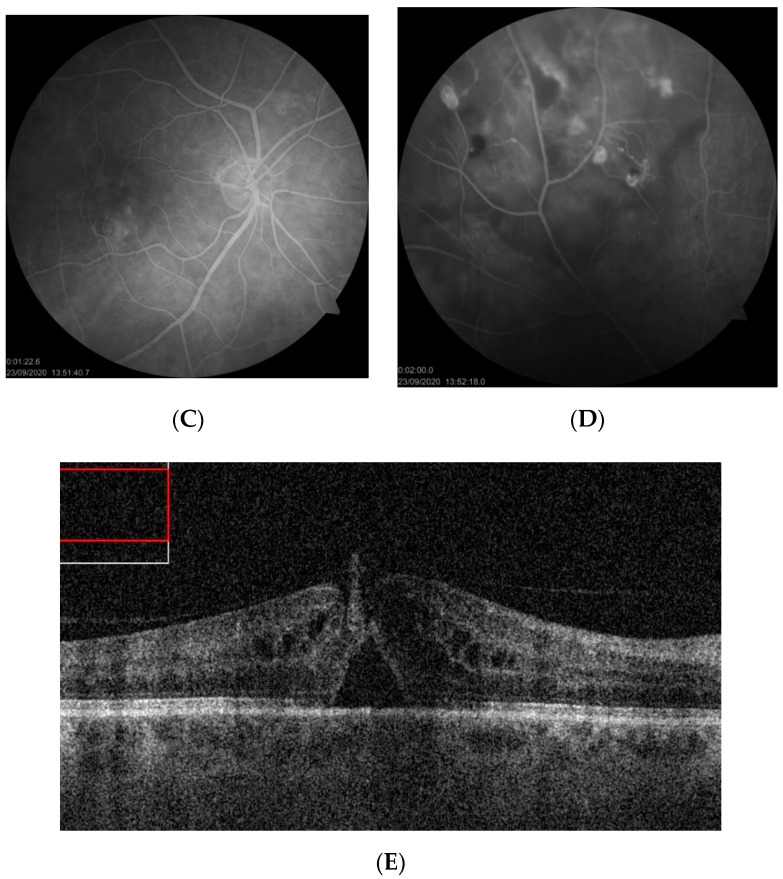
(**A**–**E**) Diagnostic images of patient 5. Fundus photographs (**A**,**B**) revealing peripheral exudates. Asteroid hyalosis obscures the visibility of the lesion. FA photos present leakage from IPT and late staining of the fovea due to CME (**C**,**D**). SD-OCT scan (**E**) confirms the presence of CME and the risk of full thickness retinal hole formation.

The last patient from category 3 (patient 5) presented with a significant decrease in BCVA to 20/100. Fundus examination and FA revealed IPT in the supratemporal quadrant with an accompanying ring of hard exudates as well as CME visible on FA in the late phase (Figure 7). SD-OCT imaging confirmed the presence of significant CME with subretinal fluid and the risk of full thickness macular hole formation (Figure 7E). Vitreoretinal traction was not observed. The patient underwent LPC applied at the peripheral lesion; however, its efficacy was limited due to asteroid hyalosis, which made the procedure difficult to perform. At the moment, the patient is scheduled for supplementary LPC and intravitreal anti-VEGF treatment. Subsequent vitreoretinal surgery is also considered with increasing risk of progression to macular hole.

### 3.3. Category 4

**Figure 8:**
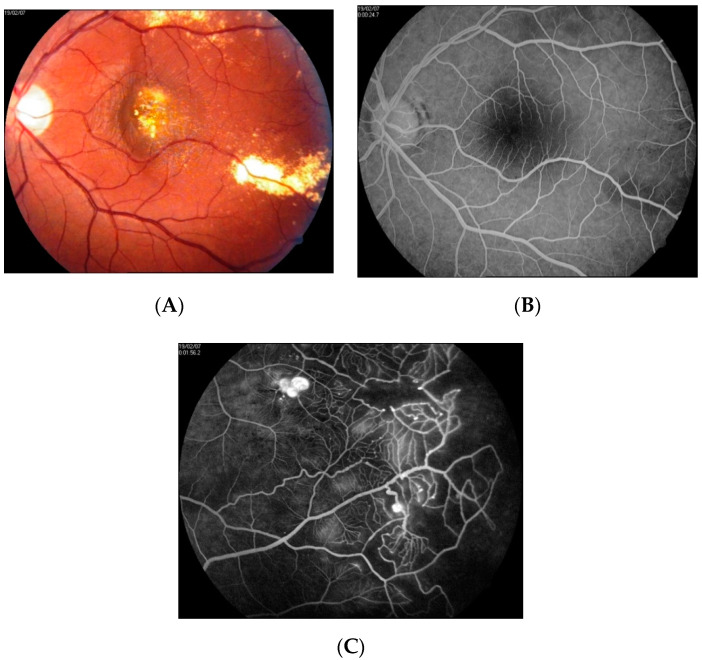
Fundus photographs (**A**) and FA images (**B**,**C**) at presentation of patient 6. A cluster of hard exudates is visible in the center of the macula. No vascular abnormalities were detected by FA at the fovea (**B**). Examination of the far periphery of the retina enabled visualization of large aneurysmal telangiectasias (**C**).

**Figure 9:**
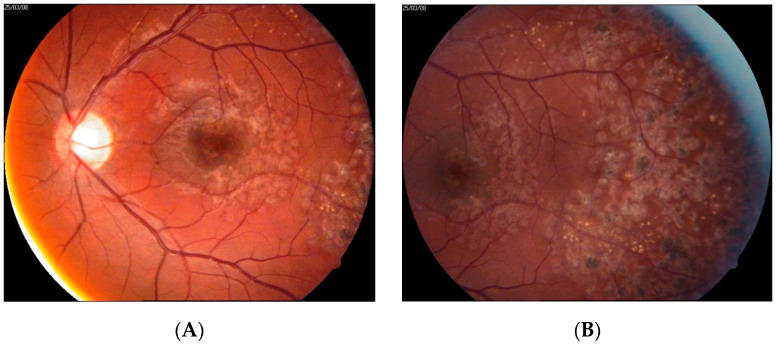

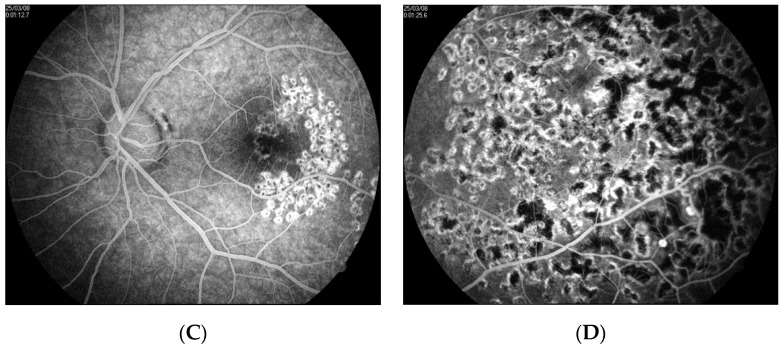
Patient 6 at one year after treatment. On the fundus photograph, complete resolution of hard exudates can be seen (**A**). Laser spots after GRID treatment in the macular center and after scatter photocoagulation at the periphery are noted (**B**). FA shows a lack of macular edema and destruction of the aneurysms at the periphery (**C**,**D**).

One patient in group 4 was diagnosed with a significant decrease in BCVA due to macular edema with the presence of hard exudates (patient 6). The course of his disease was relatively short, in that symptoms lasted between two and three weeks. The patient was diagnosed using FA only and treated with classic LPC performed in a scatter mode at the periphery and GRID at the macula. SD-OCT and intravitreal anti-VEGF therapeutics were not present on the medical market at the time of diagnosis, so were not employed for treatment purposes. Significant functional improvement was noted one month after treatment, with further improvement in the following months. Finally, after 12 months, full central vision was restored; however, some metamorphopsia persisted. Images taken at presentation and after treatment are shown in Figure 8 and Figure 9.

## 4. Literature Review

A literature search in PubMed for the term “peripheral retinal telangiectasias” and the combination of “Coats disease” and “adult onset” revealed 178 records, including 71 that referred to either adults only or both adults and children. Among these studies, there were 33 single case reports, 22 case reports of peripheral telangiectasia associated with systemic diseases, and 14 case series. Two reviews on wide-field diagnostics of peripheral telangiectasia were also found. Studies that reported at least three cases of adult-onset Coats disease published after 2000 were extracted and are presented in Table 3. The two largest population studies [10,15], which analyzed results of childhood and adult Coats disease in one cohort, are placed at the end of the table for reference.

## 5. Discussion

### 5.1. Definitions, Classification, and Presentation

IPT (or Coats disease) falls under the umbrella of the aneurysmal form of telangiectasia potentially associated with severe intraretinal leakage, subretinal exudation, and risk of retinal detachment. This phenotype differs from MACTEL type 2, which present with small telangiectatic vessels without intraretinal leakage, typical cystic appearance of the fovea, and atrophic changes in neurosensory retina [13]. The main features of IPT are capillary, venular, and arteriolar aneurysms. If located in the macular area, these aneurysms are referred to as MACTEL type 1 and are considered by some to be a variant of Coats disease [13]. Peripheral aneurysmal telangiectasias are usually larger than macular ones and more often accompanied by areas of hypoperfusion.

The presence of peripheral telangiectasias may be correlated with some systemic disorders, some of which are very rare, and, in such cases, are not referred to as Coats disease. This is an important differentiation because it influences the choice of treatment. Peripheral telangiectasia can be found in such diseases and syndromes as aplastic anemia, Bannayan-Zonaya syndrome, cutis marmorata telangiectatica congenita, multiple sclerosis, Takayasu arteritis, Cornelia de Lange syndrome, familial exudative vitreoretinopathy, genetic myopathies, dyskeratosis congenital, Coats-like retinitis pigmentosa, and intraocular tumors [23,24,25,26,27,28,29,30,31,32,33,34,35].

Variable terms were used to describe IPT, especially when they occurred in adults (Table 1). Clinicians named IPT as Coats disease (or adult-onset Coats disease in older patients), Leber miliary aneurysm, or simple peripheral retinal telangiectasia. Here, the author proposes that using a single descriptive term such as “idiopathic peripheral retinal telangiectasia” or “idiopathic peripheral aneurysmal telangiectasia” would improve communication between researchers.

In this case series, adult IPT cases were categorized into four types according to patients’ fundus presentation and foveal involvement. The proposed classification expands the well-established Shields categorization of early stages of Coats disease [10]. Shields categorized the presence of IPT without exudations as Stage 1 while Stage 2 included patients with IPT and extrafoveal exudations (2A) or foveal exudations (2B). The author suggests development of Shields Stage 2 into three categories, depending on the presence and severity of macular edema: IPT with peripheral exudations but without foveal involvement, IPT with peripheral exudations and cystoid macular edema but without macular exudates, and IPT with both peripheral and macular exudates and edema.

One must realize that Shields’ milestone report was published in 2001 when SD-OCT and intravitreal medications were not commonly in use; instead, diagnostics in Coats disease were based on fundus examination and FA results. Nowadays, however, we are able to precisely diagnose even traceable forms of macular edema with the use of SD-OCT, which makes it possible to correlate the classification of IPT with the presence and appearance of macular edema. It must further be emphasized that cases with cystoid macular edema and a lack of central exudates can be found during the course of the disease; however, they are not specifically pointed out in the Shields classification scheme. Once they are recognized and named, it will be easier to make treatment decisions, which, in contemporary ophthalmology, involve intravitreal therapies. The four forms of presentation of patients identified in the present case series may serve as a simple alternative classification system of IPT in its mild or moderate stage without retinal detachment.

### 5.2. Diagnostics

Peripheral forms of retinal telangiectasia, if asymptomatic, are probably recognized and followed quite rarely. In this case series, IPT without foveal involvement were diagnosed only after very scrupulous fundus examinations. Nevertheless, these cases could have easily been omitted by other clinicians, which probably happens most of the time with asymptomatic IPT. On the other hand, symptomatic cases with macular edema require diagnostic accuracy, precision, and inquisitiveness. If the far periphery of the retina is not examined, the presence of macular edema might be attributed to a different clinical entity, such as Irving–Gass syndrome, pars planitis, diabetic retinopathy, retinal vein occlusion, or another condition (patient 3 is an example of this). The diagnostics of peripheral retinal telangiectasia are definitely easier with the use of ultra–wide-field (UWF) FA systems, which provide an easy view of the periphery of the retina [36,37,38,39,40]. Cases with the lesions located in the far periphery might be easily missed if only standard seven field imaging is used. UWF images can also guide treatment, as the areas of abnormal vasculature and non-perfusion are visualized more precisely [39,40]. Thus, LPC or cryotherapy in IPT cases diagnosed with UWF-FA systems are potentially more effective, as all the abnormal areas of the retina are treated. [36]. Still, the use of UWF devices is not common yet due to their high cost. Nevertheless, the analysis of diagnostic processes in IPT suggests the necessity of examining the far periphery of the retina in any case presenting with an unexplained cause of macular edema.

### 5.3. Treatment

Table 3 presents the results of treatment of adult cases of IPT in the largest available studies published after 2000. Visual outcomes after treatment in general were rather poor, with a minority of patients achieving final BCVA values of greater than 20/40. The introduction of intravitreal therapies and more frequent use of lasers seemed to improve visual outcomes; however, a large percentage of patients still ended up with final BCVA values below 20/200. As can be seen, material is rather scarce, so it is difficult to formulate strong treatment recommendations based solely on experience with IPT in adult cohorts. Results of treatment of Coats disease in the pediatric population should therefore also be considered while exploring the treatment of IPT in the adult population.

Category 1 and 2 in my case series, without foveal involvement, included asymptomatic patients who were referred for FA after a routine examination of the fundus. Obviously, as mentioned earlier, many IPT cases such as these can pass unrecognized until they become symptomatic. The question of whether to treat asymptomatic patients with IPT has to be asked. Shields et al., in their first large published report [10], employed observation in 100% of stage 1 cases and in 40% of stage 2 cases (the remaining 60% were treated, including 10% with LPC and 50% with cryotherapy). The authors suggested conducting observation for mild, stationary forms of IPT without exudations. A lack of exudations in general means a lack of leakage from the telangiectasias and a small risk for subretinal fluid to occur. This is why such cases can be monitored simply during regular check-ups. In this series, one patient with such a form of IPT remained under observation without conversion into the disease’s exudative form.

Controversies might exist concerning cases with peripheral exudation but without macular edema. Yang et al., reported that macular disease in IPT progresses from the periphery to the center [41]. Shields et al., reported excellent visual outcomes in stage 1, but poor visual outcomes in 30% of cases in stage 2A of the disease [10]. More advanced stages of IPT involve larger disturbances of retinal morphology and are more difficult to treat. Large reports from the same center provide information on risk factors predictive of persistence of subretinal fluid despite treatment in stages 3–5 [42]. Poor outcome was associated with larger extent of telangiectasias and exudates, larger elevation of subretinal fluid, and the presence of iris neovascularization, and concerned 38% of patients. These data suggest that any case of progression or extension of leakage on the periphery should therefore be treated without delay, which can be done with the use of ablative procedures. Realistically, as peripheral LPC in adults is a rather uncomplicated and safe procedure that does not interfere with central vision, it can also be performed without waiting for documented progression of exudates.

The most challenging cases of IPT involve patients with accompanying macular edema (categories 3 and 4 in this series). The therapeutic approach in such cases has evolved since the advent of intravitreal injections; however, to date, there exist only a few studies that analyzed the efficacy of different treatment modalities solely in adults. This is probably due to the fact that, in most cases, Coats disease becomes symptomatic and is treated at younger ages. Adult-onset Coats disease is probably, as mentioned before, a milder form of its childhood variant, which appears to occur quite rarely. This is why a large number of cases are so hard to collect and contemporary treatment recommendations have to be based instead on case reports. The studies by Shields and colleagues constitute milestone reports of treatment in pediatric Coats disease. In 2001, Shields et al., listed the following therapeutic procedures applied in 150 cases of Coats disease (the authors report both childhood and adulthood cases together, with the median age of five years): observation, cryotherapy, LPC, and surgery for retinal detachment [10]. Meanwhile, the largest analysis so far (of 351 cases) treated over 45 years, also published by Shields et al., in 2019, reports a shift toward LPC and intravitreal therapies in the last 10 to 20 years [15].

The advent of anti-VEGF drugs apparently brought about a new chance for patients to achieve better functional results. It has been proven that VEGF levels are elevated in Coats disease and are related to disease severity, especially the amount of retinal exudations [43,44]. Therefore it has been suggested that the use of anti-VEGF agents promotes the absorption of exudates and a reduction in telangiectasias, thus improving functional outcomes [45].

Different anti-VEGF medications have been used in combination with ablative therapies in the treatment of Coats disease. A report from Bascom Palmer Institute details the successful treatment of 24 children with advanced Coats disease complicated by exudative retinal detachment with direct laser ablation in combination with anti-VEGF treatment [46]. Zhang et al., presented the results of 28 cases (including 12 adults) treated by the combination of LPC and intravitreal ranibizumab or conbercept [22], where LPC was applied after initial anti-VEGF injection and then both treatments were used in a pro re nata (PRN) fashion. These authors noted significant morphological improvements; however, the changes in BCVA were not statistically significant. On the other hand, Ramasubramanian and Shields warn that the addition of bevacizumab to standard Coats therapy (LPC and cryotherapy) might evoke vitreoretinal fibrosis and potentially retinal detachment, rarely seen after standard therapy [47]. However, Daruich et al., in a large pediatric study did not find such a relationship [48]. To date, this question remains unanswered, although clinicians emphasize the benefits of anti-VEGF treatment in Coats disease [17].

In this case series, two patients with IPT and macular edema were treated with the combination of intravitreal anti-VEGF and LPC and two cases were treated with LPC only. Interestingly, patient 6, after LPC monotherapy performed almost 15 years ago, practically had his BCVA completely restored, contrary to the results among patients treated after the advent of intravitreal therapies. I believe that this outcome is mainly due to the short duration of macular edema and aggressive LPC that was the treatment of choice at that time (as intravitreal injections were not available on the market yet). This is also consistent with the report by Smithen et al., from 2005, when LPC was also the main form of treatment of Coats disease in adults, where improvement or stabilization of BCVA was noted in 5 of 11 cases [16]. A large pediatric study from 2008 also reported that aggressive diode laser therapy proved effective in the majority of patients [49].

Among the remaining three cases of this series, one patient (patient 3) was treated with the combination of anti-VEGF and LPC, achieving morphological and functional success. The patient underwent very close follow-up and presented relatively early after symptoms occurred. Patient 4 after his initial visit was lost to follow-up for one year without direct aggressive laser treatment of IPT. During that time, severe macular edema developed and a decline in his BCVA to 20/200 was noted, and no improvements after combination treatment with anti-VEGF therapy plus LPC followed. I believe that the course of this case emphasizes the necessity of employing early ablative therapies in practically every case of symptomatic Coats disease. Only anecdotal reports of IPT well-controlled by anti-VEGF injections alone exist [50].

Photodynamic therapy has been trialed in the treatment of Coats disease; however, only a few case reports are available in the literature [51,52,53]. Thus, it should be treated as an adjunct form of treatment in refractory cases, but not as first-line therapy.

## 6. Conclusions

Adult-onset Coats disease is a clinical entity of variable presentation and extent. Available data support the use of ablative therapies, especially LPC, as the main and most effective means of treatment in every exudative form of peripheral telangiectasias. Only asymptomatic cases without exudates can be observed. Anti-VEGF treatment seems to be a useful adjunct to ablative therapies, especially in cases with macular involvement. Close follow-up is needed in every symptomatic adult-onset Coats disease case. Loss of follow-up without treatment might result in irreversible vision decline. Despite the application of different forms of therapies, a large percentage of adult Coats cases end up experiencing deterioration of vision and poor morphological outcomes.

## Figures and Tables

**Table 1:** Terminology used to describe idiopathic retinal telangiectasias.

Term	Characteristics
Coats disease	Idiopathic, nonhereditary aneurysmal retinal telangiectasia associated with intraretinal exudation and frequent exudative retinal detachment, occurring in patients aged a few months to seven decades with the peak in the first decade of life [2]
Leber miliary aneurysms	A milder variant of Coats disease, usually occurring in young adults and located at the retinal periphery [7,8,9]
Idiopathic retinal telangiectasia	A descriptive name for a Coats disease
MACTEL type 1	Macular telangiectasia type 1—aneurysmal type of macular telangiectasia, considered a central variant of Coats disease [13]
MACTEL type 2	Macular telangiectasia type 2—non-aneurysmal perifoveal capillary telangiectasia, associated with atrophy of neurosensory retina, presenting in the non-proliferative or proliferative form [13,14]

**Table 2:** Characteristics of patients with peripheral telangiectasias in this case series.

Case No/Gender	Proposed Category	Age (Years)	Eye, BCVA, Presentation of the Macula	Treatment	Follow-Up	Final BCVA and Disease State
1. M	1	20	RE, 20/20; no exudates, macular area normal	Observation	12 months	20/20; no progression
2. F	2	60	LE, 20/25; peripheral hard exudates, macular area normal	LPC in the periphery	12 months	20/25; no progression
3. M	3	20	LE, 20/60; peripheral hard exudates, CME without exudates	LPC in the periphery, intravitreal anti-VEGF	12 months	20/30; remission of CME
4. M	3	44	RE, 20/25; demarcated peripheral hard exudates, mild CME without exudates	LPC in the periphery, intravitreal anti-VEGF	20 months	20/80; progression of CME despite treatment
5. M	3	55	RE, 20/100; asteroid hyalosis, localized hard exudates outside the fovea, CME without exudates; risk of macular hole formation	LPC in the periphery	6 months	20/100; no improvement, intravitreal anti-VEGF scheduled, possible surgical treatment
6. M	4	21	LE, 6/200; peripheral and central hard exudates, CME	LPC in the periphery and GRID in the macula	12 months	20/20; remission of ME

M—male, F—female, RE—right eye, LE—left eye, BCVA—best-corrected visual acuity; CME—cystoid macular edema; LPC—laser photocoagulation; VEGF—vascular endothelial growth factor.

**Table 3:** The largest adult-onset Coats disease case series, including at least three cases and reporting treatment options, published after 2000.

Study	Population	Treatment	Mean Follow-Up	Main Outcome
Smithen et al., 2005 [16]	13 adults >35 years	LPC in 11 cases; 2 cases observed (short follow-up)	5.8 years (range: 0–17)	Average loss of 2.1 lines. BCVA improvement in 2 cases, stability in 3 cases, and decline in 6 cases. At the final follow-up, BCVA ≥ 20/40 in 5 cases and BCVA < 20/200 in 3 cases.
Goel et al., 2011 [17]	3 adults	Single intravitreal bevacizumab followed by LPC	9 months	Significant improvement of BCVA in all cases of from counting fingers to 20/300, counting fingers to 20/240, and 20/240 to 20/120; regression of hard exudates from the macula in all cases
Wang et al., 2011 [18]	3 adults	2 injections of bevacizumab followed by LPC	0.5–2 years (2, 0.5, 1 years, respectively)	Significant improvement in BCVA, reduction of CRT, and regression of telangiectasias. Case 1: BCVA change from 6/15 to 6/6.7; CRT reduction from 437 to 230 μm Case 2: BCVA change from 6/12 to 6/6, decrease in SRF (exact numbers not reported) Case 3: BCVA change from 5/60 to 6/20; CRT reduction from 412 to 330 μm
Zheng et al., 2014 [19]	5 adults	Intravitreal bevacizumab followed by LPC (3 cases) or intravitreal triamcinolone (1 case) or subsequent intravitreal bevacizumab (average of 2 injections during follow-up)	10.6 months	Resolution of subretinal fluid and telangiectasias without significant improvement in BCVA (range: 1.42–1.25 logMAR). Vitreoretinal fibrosis in two cases.
Park et al., 2016 [20]	13 adults	LPC combined with intravitreal bevacizumab (mean no. of injections: 2.69 and mean no of laser sessions: 1.68)	24.8 months	Mean BCVA change from 0.72 logMAR to 0.68 logMAR (statistically insignificant). BCVA improvement of more than 3 lines in 3 patients (23%) and stability in 7 patients (54%). Mean CRT was significantly decreased from 473 to 288 μm. Poor baseline BCVA and subfoveal hard exudates correlated with poor final BCVA result.
Rishi et al., 2016 [21]	48 adults ≥ 35 years 32 cases observed > 6 months	LPC (60.4%), observation (27.08%), surgery (6.2%), cryotherapy (4%), LPC plus cryotherapy (2%)	40 months (range: 1–122 months)	Patients with follow-up longer than 6 months (32 cases): Treated: BCVA improvement or stabilization in 82.5%; exudates reduced in 56.5%, retina attached in 95.6% Untreated: BCVA stabilization in 88%, no improvement; exudates reduced in 33%, retina attached in 66% (exact BCVA values not reported)
Zhang et al., 2018 [22]	12 adults	Intravitreal ranibizumab or conbercept followed by LPC	23.10 ± 7.8	Mean BCVA improvement significant from 1.27 ± 0.69 to 1.05 ± 0.73 logMAR; mean injection no. 2.33 ± 0.65, mean no. of laser treatments 2.5 ± 0.8
Reference studies: children and adults reported in one cohort				
Shields et al., 2001 [10]	124 eyes observed > 6 months Age 1 month to 63 years (average: 5 years)	Cryotherapy (42%), LPC (13%), observation (18%), surgery 17% and enucleation 11%	55 months (range: 6–300 months)	Anatomic improvement and stability in 76%. BCVA ≥ 20/50 in 14%, 20/60 to 20/100 in 6%, 20/200 to finger counting in 24%, and hand motion to light perception in 40%
Shields et al., 2019 [15]	351 cases, data from 45 years Age 0–79 years, median: 6 years	Overall (1973–2018): observation (21%), LPC (42%), cryotherapy (55%), sub-Tenon corticosteroids (12%), intravitreal corticosteroids (4%), anti-VEGF (10%), and primary enucleation (5%) Years 2010–2018: observation (11%), LPC (72%), cryotherapy (68%), sub-Tenon corticosteroids (29%), intravitreal corticosteroids (9%), anti-VEGF (18%), primary enucleation (1%)	58 months (range: 0–466 months)	BCVA overall Verbal >20/40 (15%) 20/50–20/200 (18%) <20/200 (48%) Preverbal Fix and follow (1%) Poor fix and follow (0.4%) No fix and follow (9%) No cooperation (9%) BCVA Years 2010–2018 Verbal >20/40 (24%) 20/50–20/200 (22%) <20/200 (40%) Preverbal Fix and follow (1%) Poor fix and follow (0.0%) No fix and follow (5%) No cooperation (6%) Disease resolution overall: 57% Disease resolution 2010–2018: 73%

BCVA—best-corrected visual acuity; CRT—central retinal thickness; LPC—laser photocoagulation; VEGF—vascular endothelial growth factor.

## Data Availability

Data is contained within the article.
